# Determination of the Absolute Configuration of Bioactive Indole-Containing Pyrazino[2,1-*b*]quinazoline-3,6-diones and Study of Their *In Vitro* Metabolic Profile

**DOI:** 10.3390/molecules26165070

**Published:** 2021-08-21

**Authors:** Solida Long, Izadora L. Furlani, Juliana M. de Oliveira, Diana I. S. P. Resende, Artur M. S. Silva, Luís Gales, José A. Pereira, Anake Kijjoa, Quezia B. Cass, Regina V. Oliveira, Emília Sousa, Madalena M. M. Pinto

**Affiliations:** 1 LQOF-Laboratório de Química Orgânica e Farmacêutica, Departamento de Ciências Químicas, Faculdade de Farmácia, Universidade do Porto, Rua de Jorge Viterbo Ferreira, 228, 4050-313 Porto, Portugal; long.solida@rupp.edu.kh (S.L.); dresende@ff.up.pt (D.I.S.P.R.); madalena@ff.up.pt (M.M.M.P.); 2 Department of Bioegineering, Faculty of Engineering, Royal University of Phnom Penh, Russian Federation Blevd, Phnom Penh 12156, Cambodia; 3 SEPARARE–Núcleo de Pesquisa em Cromatografia, Departamento de Química, Universidade Federal de São Carlos, Rodovia Washington Luiz, km 235, São Carlos 13565-905, Brazil; izadora.furlani@gmail.com (I.L.F.); juli_magalhaes_o@hotmail.com (J.M.d.O.); qcass@ufscar.br (Q.B.C.); 4 CIIMAR-Centro Interdisciplinar de Investigação Marinha e Ambiental, Terminal de Cruzeiros do Porto de Leixões, Av. General Norton de Matos S/N, Matosinhos, 4450-208 Porto, Portugal; ankijjoa@icbas.up.pt; 5 LAQV-REQUIMTE-Departamento de Química, Universidade de Aveiro, 3810-193 Aveiro, Portugal; artur.silva@ua.pt; 6 ICBAS-Instituto de Ciências Biomédicas Abel Salazar, Universidade do Porto, 4050-313 Porto, Portugal; lmgales@gmail.com (L.G.); jpereira@icbas.up.pt (J.A.P.); 7 i3S-IBMC-Instituto de Biologia Molecular e Celular, Universidade do Porto, 4050-313 Porto, Portugal

**Keywords:** gram-scale synthesis, enantioselectivity, ECD, X-ray crystallography, *in vitro* metabolism

## Abstract

In recent decades, fungi-derived naturally occurring quinazolines have emerged as potential drug candidates. Nevertheless, most studies are conducted for bioactivity assays, and little is known about their absorption, distribution, metabolism, and elimination (ADME) properties. To perform metabolic studies, the synthesis of the naturally occurring quinazolinone, fiscalin B (**1**), and its chloro derivative, 4-((1*H*-indol-3-yl)methyl)-8,10-dichloro-1-isobutyl-1,2-dihydro-6*H*-pyrazino[2,1-*b*]quinazoline-3,6(4*H*)-dione (**2**), disclosed as an antibacterial agent, was performed in a gram scale using a microwave-assisted polycondensation reaction with 22% and 17% yields, respectively. The structure of the non-natural (+)-fiscalin B was established, for the first time, by X-ray crystallography as (1*R*,4*S*)-**1**, and the absolute configuration of the naturally occurring fiscalin B (-)-**1** was confirmed by comparison of its calculated and experimental electronic circular dichroism (ECD) spectra as (1*S*,4*R*)-**1**. *in vitro* metabolic studies were monitored for this class of natural products for the first time by ultra-high-performance liquid chromatography (UHPLC) coupled with high-resolution mass spectrometry (HRMS). The metabolic characteristics of **1** and **2** in human liver microsomes indicated hydration and hydroxylation mass changes introduced to the parent drugs.

## 1. Introduction

**Figure 1:**
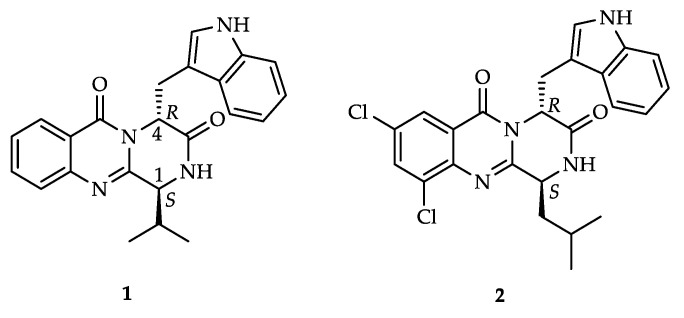
Structures of target compounds for gram syntheses, (1*S*,4*R*)-4-(1*H*-Indol-3-ylmethyl)-1-isopropyl-2*H*-pyrazino[2,1-*b*]quinazoline-3,6(4*H*)-dione (fiscalin B) (**1**) and (1*S*,4*R*)-4-[(1*H*-indol-3-yl)methyl]-8,10-dichloro-1-isobutyl-1,2-dihydro-6*H*-pyrazino[2,1-*b*]quinazoline-3,6(4*H*)-dione (**2**).

In the marine world, amino acids are important building blocks associated with diverse structures with medley complexity, particularly alkaloids from marine-derived fungi. Studies on the isolation, biosynthesis, synthesis, and biological activities of the pyrazino[2,1-*b*]quinazoline-3,6-dione core linked to an indole moiety have been increasing significantly in the last 20 years [1,2,3,4]. Structurally, these quinazolinone alkaloids have been classified from simple to complex structures and consist of three different building blocks—anthranilic acid, α-amino acids, and tryptophan moieties that merge into a simple ring system [5]. The simplest structures are glyantrypine, fumiquinazolines F and G, and fiscalin B (**1**, Figure 1), which were reported in several synthetic studies [6,7,8,9]. (1*S*,4*R*)-Fiscalin B (**1**) was first isolated in 1992 from a strain of *Aspergillus fumigatus* found in the intestine of the marine fish *Pseudolabrus japonicas* and later in 1993 from the ethyl acetate extract of a cell mass from a fungal culture of *Neosartorya fischeri* collected from We Fung Chi, Taiwan [10,11,12,13]. Naturally occurring and synthetic fiscalin B (**1**) have been reported for their activities such as substance P inhibition [12], neuroprotective [14], antitumor [15], and antimalarial [16]. However, the ADME profile of pyrazino[2,1-*b*]quinazoline-3,6-diones, including these bioactive fungi-derived compounds, has not yet been reported [1].

Owing to the increasing interest of fiscalin B (**1**) in medicinal and organic chemistry, some synthetic procedures enabling its facile production have been developed in recent decades [6,8,9,17]. Recently, we reported the synthesis and biological activities of four different series of indole-containing pyrazino[2,1-*b*]quinazoline-3,6-diones by using this synthetic methodology as well as by performing different modifications in order to study the structure–activity relationship [14,15,16,18]. From these studies, we were able to obtain a significant number of hit compounds, either as neuroprotective, antitumor, and antibacterial agents [14,15,16,18]. Herein, we report the concise total synthesis of **1**, possessing antimalarial activity [16], and its derivative 4-[(1*H*-indol-3-yl)methyl]-8,10-dichloro-1-isobutyl-1,2-dihydro-6*H*-pyrazino[2,1-*b*]quinazoline-3,6(4*H*)-dione **2**, a hit compound with antibacterial activity [18], in gram scale via a microwave-assisted methodology. This derivative exhibited potent antibacterial activity against *S. aureus* strains, with MIC values of 4 µg/mL for a reference strain (*S. aureus* ATCC 29213, positive control cefotaxime MIC = 8 µg/mL) and 8 µg/mL for a methicillin-resistant strain (*S. aureus* 66/1, positive control kanamycin MIC = 32 µg/mL). Compared with the structurally related marine natural product neofiscalin A [19], a two-fold reduction in the MIC values was observed, showing that simpler molecules are quite promising to find new agents to overcome multidrug-resistant (MDR) strains [18]. Furthermore, **2** was able to impair *S. aureus* biofilm production, and no significant cytotoxicity towards differentiated and non-differentiated SH-SY5Y cells was observed [18].

Indole-containing pyrazino[2,1-*b*]quinazoline-3,6-diones could be promising drug candidates; however, investigations on their metabolism parameters to predict biologically active metabolites or even toxicity, that could help translate these secondary metabolites to useful drug candidates and development processes, are still missing. Taking into account that the understanding and description of the metabolism of new chemical entities are an essential part of biological evaluation as an important parameter for both drug safety and efficacy, prediction of drug metabolism studies on human liver microsomes using these target alkaloids, **1** and **2**, via cytochromes P450 (phase I)-mediated reactions by UHPLC-HRMS, were performed.

## 2. Results

### 2.1. Gram-Scale Synthesis of the Enantiomeric Mixture of Fiscalin B (***1***)

**Scheme 1:**
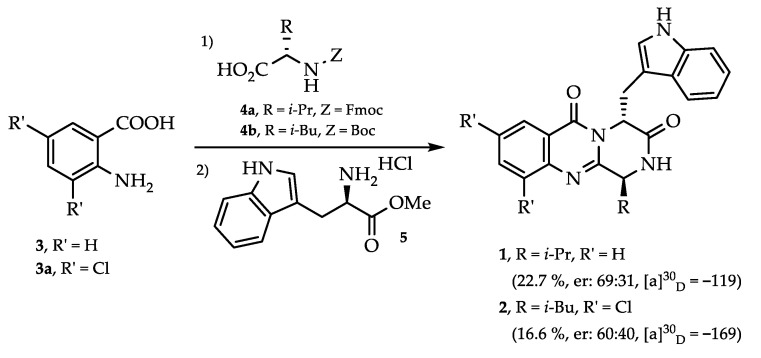
Synthesis of **1** and the derivative **2**.

The gram-scale synthesis of fiscalin B (**1**) followed the protocol used in the milligram-scale synthesis of fiscalin B (**1**) we previously reported [15]. The reaction was performed following a two-step methodology. First, *N*-Fmoc-l-valine (**4a**) and (PhO)_3_P were added to a mixture of anthranilic acid (**3**) in dry pyridine, and the reaction mixture was heated at 55 °C for 48 h, furnishing a benzoxazinone intermediate, which was not isolated. D-Tryptophan methyl ester hydrochloride (**5**) was added to the mixture, which was divided and submitted to parallel syntheses under microwave irradiation (CEM) at 220 °C under controlled power for 2 min (Scheme 1). The combined reaction mixtures were evaporated under reduced pressure to remove pyridine and dried under nitrogen. The residue was purified by silica gel flash chromatography (CH_2_Cl_2_:EtOAc:MeOH = 50:48:2), followed by preparative TLC (CH_2_Cl_2_:Me_2_CO = 95:5) to give fiscalin B (**1**) as an enantiomeric mixture in a ratio (*er*) of 69:31 and as a yellow solid (1.79 g, 22.7%) with ${\left[\alpha \right]}_{D}^{30}$ − 119 (*c* 0.045, CHCl_3_). The enantiomeric ratio (*er*) was determined by HPLC using Whelk-O1-*S*,*S* (250 mm × 4.6 mm) as a chiral column and UV detection at 245 nm. A mixture of MeOH/MeCN (50:50) was used as a mobile phase at a flow rate of 1.0 mL/min. ^1^H and ^13^C nuclear magnetic resonance (NMR) data of fiscalin B (**1**) were in accordance with those of a previous report [15].

### 2.2. Crystal Structure of Ent-(***1***)

**Figure 2:**
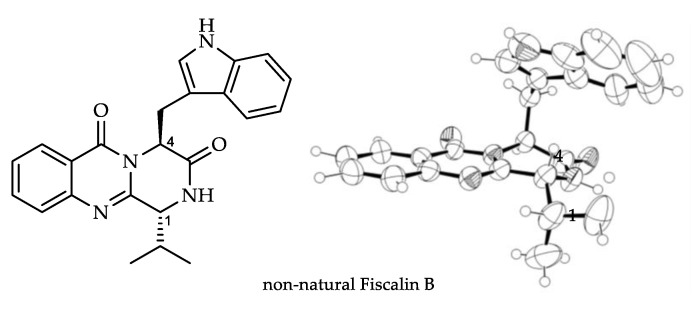
Structure of non-natural fiscalin B (1*R*,4*S*)-**1** with the Ortep view.

Unexpectedly, from the crystallization of the enantiomeric mixture of **1** from CHCl_3_ and (Me)_2_CO, a crystal form of a pure enantiomer of fiscalin B, the non-natural (1*R*,4*S*)-**1** was obtained, from which its Ortep view is presented in Figure 2. The Ortep diagram confirms that the piperazine ring is in a boat conformation. The C-4 substituent (the indolyl methyl moiety) and H-1 are in a flagpole position of the piperazine ring, while the H-4 and the C-1 substituent (*i*-Pr) are in a bowsprit position of the piperazine ring, with *N*-2 proton in an equatorial position. The optical rotation value for (1*R*,4*S*)-**1** was found to be ${\left[\alpha \right]}_{D}^{30}$ +255 (*c* 0.06, CHCl_3_).

The absolute configuration of the solid obtained from the mother liquid was identified as (1*S*,4*R*)-**1**, as determined by comparison of its calculated and experimental electronic circular dichroism (ECD) spectra, as it was not possible to obtain the compound in a suitable crystal form. The experimental ECD spectrum of (1*S*,4*R*)-**1** was measured and then compared with a quantum-mechanically simulated spectrum derived from the most significant computational models of (1*S*,4*R*)-**1** (Figure S1 in supplementary materials, see Section 3 for details). Figure S2 shows a good match between experimental and calculated spectra, with the two spectra in phase, leading to the conclusion that this compound, in fact, (1*S*,4*R*)-**1**, corresponds to natural fiscalin B (**1**).

### 2.3. Gram-Scale Synthesis of the Chloro Derivative (***2***)

Following the successful gram-scale synthesis of **1**, the synthesis of 4-[(1*H*-indol-3-yl)methyl]-8,10-dichloro-1-isobutyl-1,2-dihydro-6*H*-pyrazino[2,1-*b*]quinazoline-3,6(4*H*)-dione (**2**), reported as the most potent antimicrobial derivative from a library of indole-containing pyrazino[2,1-*b*]quinazoline-3,6-diones [18], was attempted using a microwave-assisted procedure. The condensation of 3,5-dichloroanthranilic acid (**3a**) with *N*-Boc-l-leucine (**4b**, Scheme 1) and D-tryptophan methyl ester hydrochloride (**5**) proved to be successful to give **2** as a yellow solid (0.78 g, 16.6% from 0.01 mol of the starting materials). The yield of this gram scale of **2** was higher when compared to the milligram scale, probably due to the prolonged reaction time of the first condensation and with the increased amount of the coupling agent from 1.2 to 1.5 equiv. This increase in the yield was also observed in the gram-scale synthesis of **1**. The enantiomeric ratio of **2** was found to be 3:2 using the same HPLC conditions as described for **1**.

The structure of **2**, elucidated by ^1^H and ^13^C NMR spectroscopy, was in accordance with that previously reported [18]. Due to the effect of the two chlorine substituents at C-9 and C-11, the signals corresponding to H-8 and H-10 of the anthranilic acid moiety of **2** appeared as narrow doublets at δ_H_ 8.13 (*J* = 2.4 Hz) and 7.75 (*J* = 2.4 Hz), respectively. The splitting of the H-1′ of L-leucine moiety in **2** into two different δ values confirms the presence of diastereotopic protons. The signal corresponding to H-2 appeared as a broad singlet at δ_H_ 7.14 (DMSO-d_6_) while for H-1 appeared as a double doublet at δ_H_ 2.68 (*J* = 7.3, 4.9 Hz). These assignments revealed that **2** corresponds to the *trans*-isomer [18].

### 2.4. In vitro Metabolism in Human Liver Microsomes

Drug metabolism studies of new chemical entities (NCEs) play an essential role in the early phase of drug discovery and development programs, as the metabolites generated could present beneficial therapeutic efficacy or lead to serious toxicological effects [20]. Metabolic profiling studies to identify metabolites generated from human liver cytochrome P450 (CYP) microsomes, the primary enzyme system involved in phase I metabolism, were used for *in vitro* biotransformation studies of the enantiomeric mixture **1** and its chloro derivative **2**.

The major phase I reactions include hydroxylation, oxidation, and hydrolysis. Human liver microsomes (HLMs) are the most widely used for drug metabolism studies, as they are a good alternative to the in vivo human metabolism studies due to ethics and safety issues in the early drug discovery stage. In addition, HLMs are commercially available and promote great contributions to predict in vivo outcomes from *in vitro* data [21,22]. Phase I reactions were investigated by using UHPLC-HRMS data screening and processing, which can provide accurate mass, elemental composition, and the error (ppm) between calculated mass and measured mass [23]. Besides HRMS data, the annotated metabolites were also estimated in silico by MetabolitePredict^®^ Software (Bruker Daltonics, version 2.0). All detected metabolites were analyzed using data-dependent acquisition (time-of-flight (TOF)-MS/MS) in a positive ion mode to elucidate the molecular structures.

The MS^2^ fragmentation profile of fiscalin B (**1**) and its chloro derivative (**2**) was initially acquired and then used as a control to facilitate the structure identification of the *in vitro* metabolism products. The ions that were absent in the control samples were considered metabolites. Fiscalin B (**1**) showed a protonated molecular ion [M + H]^+^ at *m*/*z* 387.1814 (retention time (R_t_) = 4.64 min) and produced four characteristic fragment ions at *m*/*z* 258.1240, *m*/*z* 214.0613, *m*/*z* 170.0597, and *m*/*z* 130.0655 that were used as diagnostic ions for the metabolites formed. The fragment ions at *m/z* 258.1240 and 214.0613 were attributed to the loss of the indole moiety [C_9_H_7_N] and a neutral loss of propane [C_3_H_8_], respectively. The fragment ion at *m/z* 170.0597 was likely generated from the cleavage of the C-N bond, derived from the protonated ion *m/z* 387.1814. The UHPLC-HRMS/MS spectra of fiscalin B (**1**) and its proposed fragmentation products are shown in Figures S3 and S4.

The *in vitro* metabolism of **1** furnished three possible metabolites. The difference in analysis between blank samples and the **1**-containing incubation sample was performed by MetaboliteTools^®^ software (Bruker Daltonics, Billerica, MA, CA, USA). The most abundant molecular ion at *m*/*z* 403.1753 (R_t_ 3.73 min) was designated as M1, corresponding to [C_23_H_23_N_4_O_3_]^+^_._ The fragment ions at *m*/*z* 256.1080 and *m*/*z* 214.0606 imply that the pyrazino[1,2-*b*]quinazoline-3,6-dione moiety remained unmodified, while the fragment ions at *m*/*z* 186.0593 and *m*/*z* 146.0599 were 16 *amu* higher than those of the parent ion, suggesting a modification by hydroxylation reaction in the indole moiety (Figures S5 and S6). The molecular ion at *m*/*z* 419.1708 (R_t_ 2.56 min) was attributed to metabolite M2, corresponding to [C_23_H_23_N_4_O_4_]^+^. It was detected with an accurate mass of 32 *amu* higher than that of the parent ion, suggesting that fiscalin B (**1**) was likely modified by two hydroxylation reactions. The fragment ion at *m*/*z* 401.1623 implies a neutral loss of water (−18 *amu*) caused by hydroxylation of the isobutyl moiety. The fragment ions observed at *m*/*z* 186.0632 and *m*/*z* 146.0596 suggest that the second hydroxylation occurred in the indole moiety of the M1 metabolite (Figures S7 and S8).

The annotated metabolite M3 showed an accurate protonated molecular ion at *m*/*z* 421.1863 (R_t_ 2.41 min) and a molecular formula of C_23_H_24_N_4_O_4_. According to the molecular ion and fragment ions spectrum, it can be inferred that hydroxylation in the isobutyl moiety and hydrolysis in the C-6 position occurred. The ion fragment at *m*/*z* 403.1804 evidences a neutral loss of water (−18 *amu*) reconstituting again the pyrazino[1,2-*b*]quinazoline-3,6-dione moiety. The fragment ion at *m*/*z* 274.1178, suggests the loss of the indole moiety, also implying that the second hydroxylation reaction occurred in the isobutyl group. Unlike M1 and M2, the indole moiety of the M3 metabolite remained intact, showing the fragment ions at *m*/*z* 170.0806 and *m*/*z* 130.0653. Figures S9 and S10 illustrate the fragment ions spectrum and the fragmentation pathways for M3, respectively.

**Figure 3:**
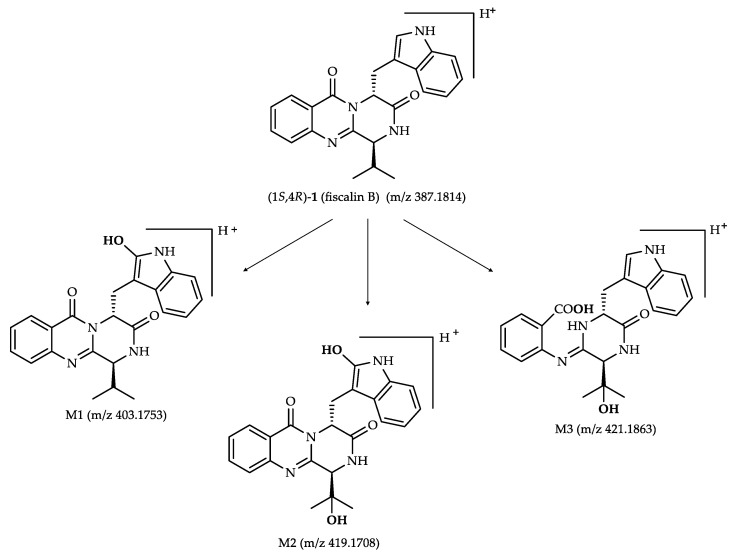
Inferred *in vitro* metabolites of **1**.

The metabolites produced by the chloro derivative (**2**) were inferred in a similar manner. The UHPLC-HRMS/MS spectrum of the chloro derivative (**2**) showed a protonated molecular ion at *m*/*z* 469.1181 (R_t_ 5.56 min)*,* with three characteristic fragment ions at *m*/*z* 338.0453, *m*/*z* 170.0595, and *m*/*z* 130.0653, suggesting similar losses to those observed for fiscalin B (**1**); however, in this case, the *m*/*z* 338.0453 ion corresponds to pyrazino[1,2-*b*]quinazoline-3,6-dione with two chlorine substituents at C-9 and C-11. The UHPLC-HRMS/MS spectrum and the fragmentation pathways of the chloro derivative (**2**) are shown in Figures S11 and S12. The detailed characterization of the *in vitro* metabolites of **1** is presented in Table 1, and the inferred *in vitro* metabolites are described in Figure 3.

The *in vitro* metabolism of the chloro derivative (**2**) resulted in two metabolites. Metabolite M4, corresponding to [C_24_H_23_Cl_2_N_4_O_3_]^+^ showed a molecular ion at *m*/*z* 485.1137 (R_t_ 4.85 min) with the highest abundance. An increase of 16 *amu* relative to the parent compound infers a hydroxylation reaction in the indole moiety. The fragmentation of M4 generates a fragment ion that corresponds to the indole group at *m*/*z* 146.0585 and a second fragment ion at *m*/*z* 340.0616, which corresponds to the 9,11-dichloro-pyrazino[1,2-*b*]quinazoline-3,6-dione moiety (Figures S13 and S14). A second metabolite, designated as M5, is consistent with the molecular formula [C_24_H_23_Cl_2_N_4_O_4_]^+^ and a molecular ion at *m*/*z* 501.1105 (R_t_ 3.73 min). According to the molecular ion and fragment ions spectrum, it can be inferred that two hydroxylations have occurred to produce metabolite M5, one at the C-C bond between the indole and pyrazino[1,2-*b*]quinazoline-3,6-dione moieties and the other in the indole moiety. The fragment ions suggest the loss of the hydroxylated indole moiety generating the fragment ion at *m*/*z* 162.0549 and the residual part of the molecule generates the fragment ion at *m*/*z* 340.0617.

**Figure 4:**
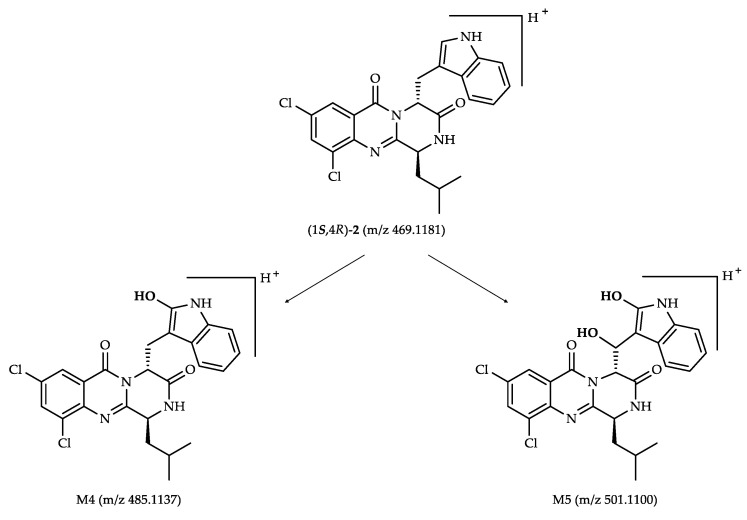
Inferred *in vitro* metabolites of **2**.

The detailed characterization of the *in vitro* metabolites of **2** is presented in Table 2, and the inferred *in vitro* metabolites are described in Figure 4.

## 3. Materials and Methods

### 3.1. Synthesis

#### 3.1.1. Materials and Methods

All reagents were analytical grade. Dried pyridine and triphenylphosphite were purchased from Sigma (Sigma-Aldrich Co. Ltd., Gillingham, UK). Anthranilic acid (**3**) and derivative **3a**, protected amino acids **4a** and **4b**, and tryptophan (**5**) were purchased from TCI (Tokyo Chemical Industry Co. Ltd., Chuo-ku, Tokyo, Japan). Column chromatography purifications were performed using flash silica Merck 60, 230–400 mesh (EMD Millipore Corporation, Billerica, MA, USA). Preparative TLC was carried out on pre-coated Merck Kieselgel 60 F_254_ plates (EMD Millipore Corporation, Billerica, MA, USA), and spots were visualized under UV light (Vilber Lourmat, Marne-la-Vallée, France). Melting points were measured in a Kofler microscope and uncorrected. Infrared spectra were recorded in a KBr microplate in an FTIR spectrometer Nicolet iS10 from Thermo Scientific (Waltham, MA, USA) with Smart OMNI-Transmission accessory (Software 188 OMNIC 8.3, Thermo Fisher Scientific Inc., Austin, TX, USA). ^1^H and ^13^C NMR spectra were recorded in CDCl_3_ (Deutero GmbH, Kastellaun, Germany) at room temperature unless otherwise mentioned on a Bruker AMC instrument (Bruker Biosciences Corporation, Billerica, MA, USA) operating at 300 MHz for ^1^H and 75 MHz for ^13^C. Carbons were assigned according to DEPT, HSQC, and/or HMBC experiments. Optical rotations were measured at 25 °C with an ADP 410 polarimeter (Bellingham + Stanley Ltd., Tunbridge Wells, Kent, UK) using the emission wavelength of a sodium lamp. Concentrations are given in g per 100 mL. HRMS were measured on a Bruker FTMS APEX III mass spectrometer (Bruker Corporation, Billerica, MA, USA) recorded as ESI (Electrospray) made in Centro de Apoio Científico e Tecnolόxico à Investigation (CACTI, University of Vigo, Pontevedra, Spain).

#### 3.1.2. Gram-Scale Synthesis of Enantiomeric Mixture of Fiscalin B (**1**)

In a closed, two-neck, round-bottomed flask, anthranilic acid (**3**, 4.2 g, 0.03 mol), *N*-Fmoc-l-valine (**4a**, 10.4 g, 0.03 mol), and triphenyl phosphite (11.81 mL, 0.045 mol) were added along with dry pyridine (150 mL). The flask was heated in a heating block with stirring at 55 °C for 48 h. After cooling the mixture to a room temperature, D-tryptophan methyl ester hydrochloride (**5**, 7.65 g, 0.03 mol) was added, and the reaction mixture was divided into 5 mL mixtures in 30 microwave vials that were further irradiated in the microwave at a constant temperature (220 °C) for 2 min in parallel. The reactions were gathered, and the work-up and purification were performed as described in a previous report [15]. Compound **1** was collected as a yellow solid. Yield: 1.79 g, 22.7%; mp: 168–169 °C; er = 69:31; ${\left[\alpha \right]}_{D}^{30}$ −119 (*c* 0.045; CHCl_3_); IR *v_max_*: ^1^H NMR and ^13^C NMR data, and (+)-HRMS-ESI (See in [15]).

### 3.2. X-ray Crystallography

The powder enantiomeric mixture of **1** was subjected to crystallization from a mixture of CHCl_3_ and (Me)_2_CO. The light-yellow cubical crystals of non-natural fiscalin B (+)-**1** were obtained after recrystallization of **1** from a mixture of CHCl_3_ and (Me)_2_CO and washed several times with CHCl_3_.Yield: 0.253 g, 3.3% mp: 169–169.3 °C; ${\left[\alpha \right]}_{D}^{30}$ + 255 (*c* 0.06; CHCl_3_); IR *v_max_*, ^1^H NMR and ^13^C NMR data (See in [16]).

The crystal of (+)-**1** was mounted on a cryoloop using paratone. X-ray diffraction data were collected at room temperature with a Gemini PX Ultra equipped with CuK_α_ radiation (λ = 1.54184 Å). The structure was solved by direct methods using SHELXS-97 and refined with SHELXL-97 [24]. Crystal was monoclinic, space group P2_1_/c, cell volume 2047.89(15) Å^3^, and unit cell dimensions *a* = 18.0056(9) Å, *b* = 9.3592(3) Å and *c* = 12.7126(6) Å and β = 107.074(5) (uncertainties in parentheses). Non-hydrogen atoms were refined anisotropically. Hydrogen atoms were either placed at their idealized positions using appropriate HFIX instructions in SHELXL and included in subsequent refinement cycles or were directly found from different Fourier maps and were refined freely with isotropic displacement parameters. The refinement converged to R (all data) = 12.46% and wR2 (all data) = 29.12%.

### 3.3. Electronic Circular Dichroism (ECD)

The experimental ECD spectrum was carried out from the mother liquid of **1** (10 mmol L^−1^ in acetonitrile) obtained in a Jasco J-815 CD spectropolarimeter with a 0.1 mm cuvette and 8 accumulations. The simulated ECD spectrum was obtained by first determining all the relevant conformers of the (1*S*,4*R*)-**1** computational model. These were constructed from an MM2 minimized conformation (with the piperazine ring in boat conformation) by rotating the bonds C-1/C-1′, C-4/C-4′, and C-4′/C-5′ in steps of 120°, 120°, and 180°, respectively. The resulting 18 conformers were minimized in Gaussian 16W (Gaussian Inc., Wallingford, CT 06492, USA) using the DFT B3LYP method with a 6-31 G basis set in conjunction with an IEFPCM solvation model of acetonitrile. The five most stable conformers, accounting for 97% of the Boltzmann-weighted population, were further minimized using the more accurate B3LYP/6-311 + G(2d,p)/IEFPCM method. Its first 70 ECD transitions were then calculated by using the same method, coupled with the TD method for excited states calculation. The line spectrum for each one of the five conformations was built by applying a Gaussian line broadening of 0.2 eV to each computed transition with a constant UV shift of 6 nm. The final ECD spectrum was obtained by the Boltzmann-weighted sum of the five-line spectra [25].

### 3.4. General Procedure of the Gram-Scale Synthesis of 4-((1H-Indol-3-yl)methyl)-8,10-dichloro-1-isobutyl-1,2-dihydro-6H-pyrazino[2,1-b]quinazoline-3,6(4H)-dione (***2***)

In a closed, two-neck, round-bottomed flask, 3,5-dichloroanthranilic acid (**3a**, 2.06 g, 0.01 mol), *N*-Boc-l-leucine (**4b**, 2.3 g, 0.01 mol), and triphenyl phosphite (3.9 mL, 0.015 mol) were added along with dry pyridine (50 mL), and the reaction mixture was stirred at 55 °C for 48 h. After cooling to a room temperature, D-tryptophan methyl ester hydrochloride (**5**, 2.55 g, 0.01 mol) was added, and the reaction mixture was divided into 5 mL mixtures in 30 microwave vials that were irradiated in the microwave at a constant temperature (220 °C) for 2 min in parallel. After removing the solvent with toluene, the crude product was purified by flash column chromatography using *n*-hexane/EtOAc (60:40) as a mobile phase. Afterwards, preparative TLC was performed using CH_2_Cl_2_/Me_2_CO (95:5) as a mobile phase. The major compound showed no fluorescence under UV light. Compound **2** was collected as a yellow solid. Yield: 0.78 g, 16.62%; er = 60:40; mp: 254.7–255.1 °C; ${\left[\alpha \right]}_{D}^{30}$ −169 (*c* 0.056; CHCl_3_); ^1^H NMR and ^13^C NMR data, and (+)-HRESIMS are in accordance with previously published data [18].

### 3.5. In vitro Metabolism Assay

Before carrying out the metabolism experiments, **1** and **2** were re-purified by semi-preparative liquid chromatography (LC) using a Luna^®^ C18 column (250 × 7.0 mm; 10 µm) and isocratic elution mode using methanol and water (60:40) as a mobile phase and a flow rate of 1 mL min^−1^. The semi-preparative LC was performed using a Shimadzu LC-system (Shimadzu, Kyoto, Japan) equipped with two LC-20AD pumps, an SIL-20A auto-injector, an SPD-20A UV-vis detector, a CBM-20A controller, and a Lab Solutions^®^ software (version 1.25).

The *in vitro* metabolism assays were performed using human liver microsomes from 50 different individual donors (Thermo Fisher Scientific, San Francisco, CA, USA). Aliquots of HLMs were thawed on ice. A solution containing 2.59 mmol L^−1^ of **1** was prepared in acetonitrile and diluted with ammonium acetate (15 mmol L^−1^, pH 7.4) to a final concentration of 20 µmol L^−1^. Then, this solution was pre-incubated for 5 min at 36 °C in a mixture containing 175 µL of ammonium acetate buffer (15 mmol L^−1^, pH 7.4) and 25 µL of human liver microsomes 10 mg mL^−1^ of protein. The reaction was started by adding 25 µL of NADPH 10 mmol L^−1^. The mixture was placed at a constant temperature of 36 °C, and an aliquot of 100 µL was extracted after 60 min. The reaction was terminated by adding 80 µL of acetonitrile, followed by centrifugation for 10 min at 7267 g. The supernatant was transferred to a vial for LC-HRMS/MS analysis. The blank sample was prepared from the reaction at time 0 min. The same protocol was used for **2**.

### 3.6. LC-HRMS/MS Analysis

The *in vitro* metabolism experiments were monitored with an HRMS containing a quadrupole time-of-flight mass analyzer (QTOF). MS analysis was performed using an Impact HD QTOF™ mass spectrometer (Bruker Daltonics, Bremen, Germany) equipped with an electrospray ionization (ESI) interface operating in a positive ion mode.

The optimal parameters were set as follows: positive ion mode, capillary voltage, 3600 V; end plate offset, 450 V; nebulizer, 4 bar; dry gas flow, 8 L min^−1^; dry heater temperature, 180 °C; collision cell energy, 5 eV, and full MS scan range, *m/z* 100–1100. The mass spectrometer was programmed to perform acquisition in data-dependent acquisition (DDA) MS/MS mode using a dynamic method of 3 s of cycle time, release after 0.9 min, and exclude *m/z* after 1 spectrum. MS spectra rate of 2.00 Hz and MS/MS spectra rate of 2.00 Hz for low precursor ions (50,000 cts.) and 4.00 Hz for high precursor ions (100,000 cts). The experiments used a range of collision energy from 25 until 62.5 eV for all *m*/*z* ranges analyzed.

Chromatographic separation of **1** metabolism was carried out with a Phenomenex^®^ Phenyl-Hexyl column (50 × 2.1 mm; 5 µm). The mobile phase consisted of 0.1% formic acid either in water (solvent A) or acetonitrile (solvent B). A linear gradient was set as follows: 20–95% B, 0–8 min; 95–20% B, 8.01–11 min. The flow rate was 0.2 mL min^−1^, and the sample injection volume was 5 μL. For **2**, the same condition was applied, following a linear gradient of 30–95% B, 0–8 min; 95–20% B, 8.01–11 min.

Internal mass-spectrometer calibration was performed with 1 mmol L^−1^ of sodium formate prepared in acetonitrile, using the quadratic high-precision calibration (HPC) regression mode. The calibration solution was injected at the end of the analytical run (8.01–9 min) and all the spectra were calibrated before compound identifications.

For structural elucidation of the metabolites, the analyses were acquired in MS/MS mode and the fragments spectra were compared to those of **1** and **2**. MetabolitePredict^®^ Software was also used as a tool for the in silico prediction of metabolites.

## 4. Conclusions

In summary, we described, for the first time, a gram-scale synthesis using parallel reactions of quinazolinone **1** and its antimicrobial derivative **2**. We also reported for the first time the crystal structure of (1*R*,4*S*)-**1** (non-natural). These syntheses constitute an expeditious approach to this family of natural products with potential application to large-scale compound production for further biological/pharmacological assays. In the present study, the produced alkaloids, **1** (fiscalin B) and its chloro derivative (**2**), were further used for the identification of phase I metabolites obtained from human liver microsomes. A total of three metabolites were detected for **1** (M1, M2, and M3) and two for **2** (M4 and M5). The metabolites were inferred by their retention time, accurate masses, and fragment ions. Additionally, the metabolic studies provided preliminary and valuable information in predicting metabolites from the human microsome that could improve the safety profile of these compounds as drug candidates.

## Figures and Tables

**Table 1:** Characterization of *in vitro* metabolites of fiscalin B (**1**) by UHPLC-HRMS/MS.

Peak ID	Formula	R_t_ (min)	*m*/*z*	Error (ppm)	Nominal Mass Change (Da)
Parent	C_23_H_23_N_4_O_2_	4.6	387.1816	0.0	-
M1	C_23_H_23_N_4_O_3_	3.7	403.1764	0.1	+16
M2	C_23_H_23_N_4_O_4_	2.6	419.1718	−0.9	+32
M3	C_23_H_25_N_4_O_4_	2.4	421.1863	+1.8	+34

**Table 2:** Characterization of *in vitro* metabolites of **2** by UHPLC-HRMS/MS.

Peak ID	Molecular Formula	R_t_ (min)	*m*/*z*	Error (ppm)	Nominal Mass Change (Da)
Parent	C_24_H_23_Cl_2_N_4_O_2_	5.56	469.1188	1.0	-
M4	C_24_H_23_Cl_2_N_4_O_3_	4.85	485.1136	1.2	+16
M5	C_24_H_23_Cl_2_N_4_O_4_	3.73	501.1105	−2.8	+32

## Data Availability

The data presented in this study are available in this article and respective supplementary information.

## Supplementary Materials

Figure S1. Model of the most abundant conformation of 1 (B3LYP/6-311 + G(2d,p) lowest energy conformer accounting for 51% of conformer population) in its (1S,4R) configuration, as assigned by ECD; Figure S2. Experimental ECD spectrum (solid line, left axis) of (−)-1 and theoretical ECD spectrum (dotted line, right axis) of (1S,4R)-1 configuration; Figure S3. UHPLC-HRMS/MS spectrum of fiscalin B (**1**), *m/z* 387.1826*;* Figure S4. Proposed fragmentation pathways of fiscalin B in positive ion mode*;* Figure S5. UHPLC-HRMS/MS spectrum of M1, *m/z* 403.1765*;* Figure S6. Proposed fragmentation pathways of metabolite M1*;* Figure S7. UHPLC-HRMS/MS spectrum of M2, *m/z* 419.1708*;* Figure S8. Proposed fragmentation pathways of metabolite M2*;* Figure S9. UHPLC-HRMS/MS spectrum of M3, *m/z* 421.1863*;* Figure S10. Proposed fragmentation pathways of metabolite M3*;* Figure S11. UHPLC-HRMS/MS spectrum of fiscalin B chloro derivative (**2**), *m/z* 469.1193 in positive ion mode*;* Figure S12. Proposed fragmentation pathways of fiscalin B chloro derivative (**2**)*;* Figure S13. UHPLC-HRMS/MS spectrum of M4, *m/z* 485.1146*;* Figure S14. Proposed fragmentation pathways of metabolite M4*;* Figure S15. UHPLC-HRMS/MS spectrum of M5, *m/z* 501.1105*;* Figure S16. Proposed fragmentation pathways of metabolite M5.

## Sample Availability

Samples of compounds **1** and **2** are available from the authors.
