# Supplementary material for: Determination of the Absolute Configuration of Bioactive Indole-Containing Pyrazino[2,1-b]quinazoline-3,6-diones and Study of Their In Vitro Metabolic Profile
Source: Molecules. 2021 Aug 21;26(16):5070. doi: 10.3390/molecules26165070 (PMC8398919; doi:10.3390/molecules26165070)
Supplement: Supplementary file 1 [file molecules-26-05070-s001.zip › molecules-1340208-supplementary.pdf]

Determination of the absolute configuration of bioactive indole-containing pyrazino[2,1-*b*]quinazoline-3,6-diones and study of their *in vitro* metabolic profile

Solida Long [1,2,†], Izadora L. Furlani [3,†], Juliana M. de Oliveira [3], Diana I. S. P. Resende [1,4], Artur M. S. Silva [5], Luís Gales [6,7], José A. Pereira [6], Anake Kijjoa [4,6], Quezia B. Cass [3], Regina V. Oliveira [3,*], Emília Sousa [1,4,*], Madalena M. M. Pinto [1,4]

[1] LQOF - Laboratório de Química Orgânica e Farmacêutica, Departamento de Ciências Químicas, Faculdade de Farmácia, Universidade do Porto, Rua de Jorge Viterbo Ferreira, 228, 4050-313 Porto, Portugal; up201502099@ff.up.pt (S.L.); dresende@ff.up.pt (D.I.S.P.R.); esousaf.up.pt (E.S.); madalena@ff.up.pt (M.M.M.P.)
[2] Department of Bioegineering, Faculty of Engineering, Royal University of Phnom Penh, Russian federation Blevd. Phnom Penh, Cambodia; long.solida@rupp.edu.kh (S.L)
[3] *SEPARARE* – Núcleo de Pesquisa em Cromatografia, Departamento de Química, Universidade Federal de São Carlos, Rodovia Washington Luiz, km 235, 13565-905 São Carlos, Brazil; izadora.furlani@gmail.com (I.L.F.); juli_magalhaes_o@hotmail.com (J.M.de O); qcass@ufscar.br (Q.B.C.); oliveirarv@ufscar.br (R.V.O.)
[4] CIIMAR - Centro Interdisciplinar de Investigação Marinha e Ambiental, Terminal de Cruzeiros do Porto de Leixões, Av. General Norton de Matos S/N, 4450-208 Matosinhos, Porto, Portugal.
[5] LAQV-REQUIMTE - Departamento de Química, Universidade de Aveiro, 3810-193 Aveiro, Portugal; artur.silva@ua.pt (A.M.S.S.)
[6] ICBAS - Instituto de Ciências Biomédicas Abel Salazar, Universidade do Porto, Portugal; ankijjoa@icbas.up.pt (A.K.); jpereira@icbas.up.pt (J.A.P.)
[7] i3S-IBMC-Instituto de Biologia Molecular e Celular, Universidade do Porto, Porto, Portugal; lmgales@gmail.com (L.G.)
* Correspondence: esousa@ff.up.pt; Tel.: +351 220 428 689; oliveirarv@ufscar.br; Tel: +55-16- 3306-6837
† Both authors contributed equally to this work

Content

Figure S1. Model of the most abundant conformation of 1 (B3LYP/6-311+G(2d,p) lowest energy conformer accounting for 51% of conformer population) in its (1S,4R) configuration, as assigned by ECD. 2
Figure S2. Experimental ECD spectrum (solid line, left axis) of (-)-1 and theoretical ECD spectrum (dotted line, right axis) of (1S,4R)-1 configuration. 2
Figure S3. LC-HRMS/MS spectrum of fiscalin B (**1**), *m/z* 387.1826. 3
Figure S4. Proposed fragmentation pathways of fiscalin B (**1**) in positive ion mode. 3
Figure S5. LC-HRMS/MS spectrum of M1, *m/z* 403.1765. 4
Figure S6. Proposed fragmentation pathways of metabolite M1. 4
Figure S7. LC-HRMS/MS spectrum of M2, *m/z* 419.1708. 5
Figure S8. Proposed fragmentation pathways of metabolite M2. 5
Figure S9. LC-HRMS/MS spectrum of M3, *m/z* 421.1863. 6
Figure S10. Proposed fragmentation pathways of metabolite M3. 6
Figure S11. LC-HRMS/MS spectrum of fiscalin B chloro derivative (**2**), *m/z* 469.1193. 8
Figure S12. Proposed fragmentation pathways of fiscalin B chloro derivative (**2**) in positive ion mode. 8
Figure S13. LC-HRMS/MS spectrum of M4, *m/z* 485.1146. 9
Figure S14. Proposed fragmentation pathways of metabolite M4. 9
Figure S15. LC-HRMS/MS spectrum of M5, *m/z* 501.1105. 10
Figure S16. Proposed fragmentation pathways of metabolite M5. 10

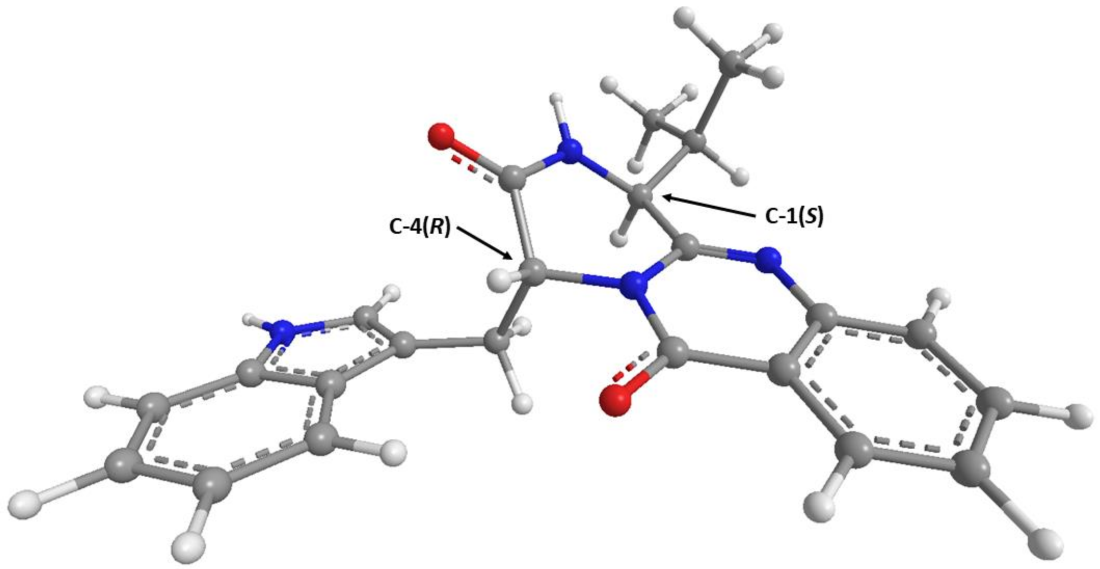

Figure S1. Model of the most abundant conformation of 1 (B3LYP/6-311+G(2d,p) lowest energy conformer accounting for 51% of conformer population) in its (1S,4R) configuration, as assigned by ECD.

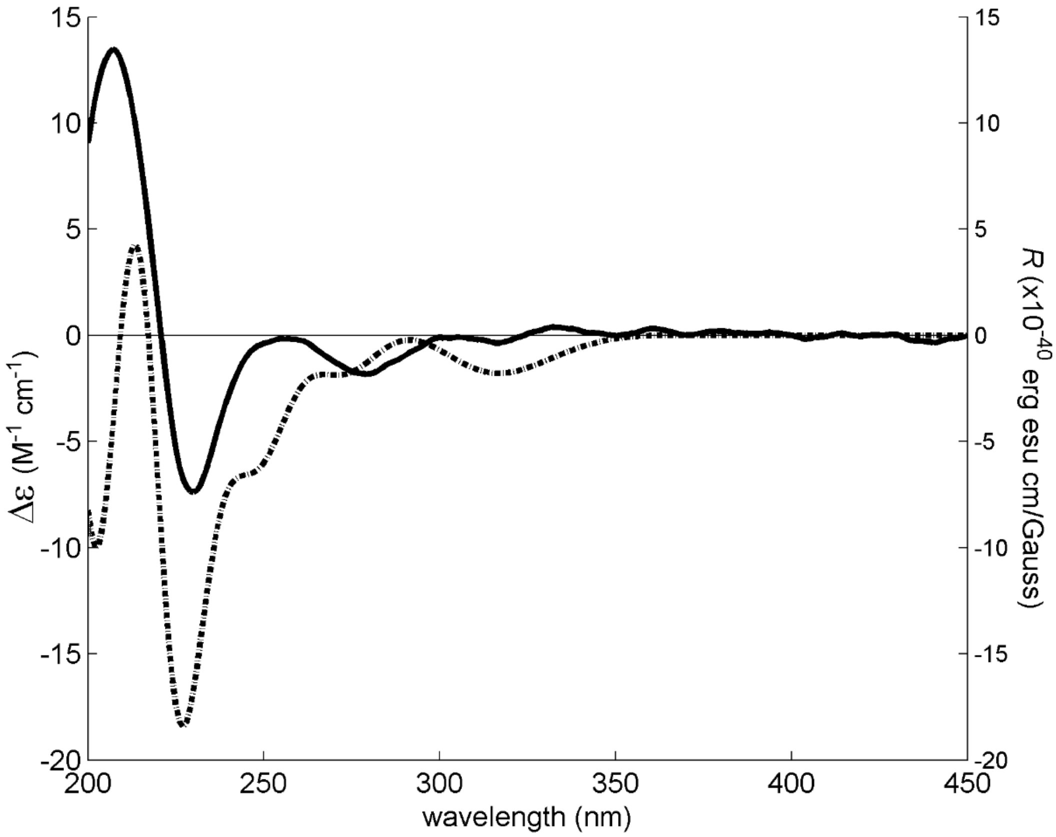

Figure S2. Experimental ECD spectrum (solid line, left axis) of (-)-**1** and theoretical ECD spectrum (dotted line, right axis) of (1S,4R)-**1** configuration.

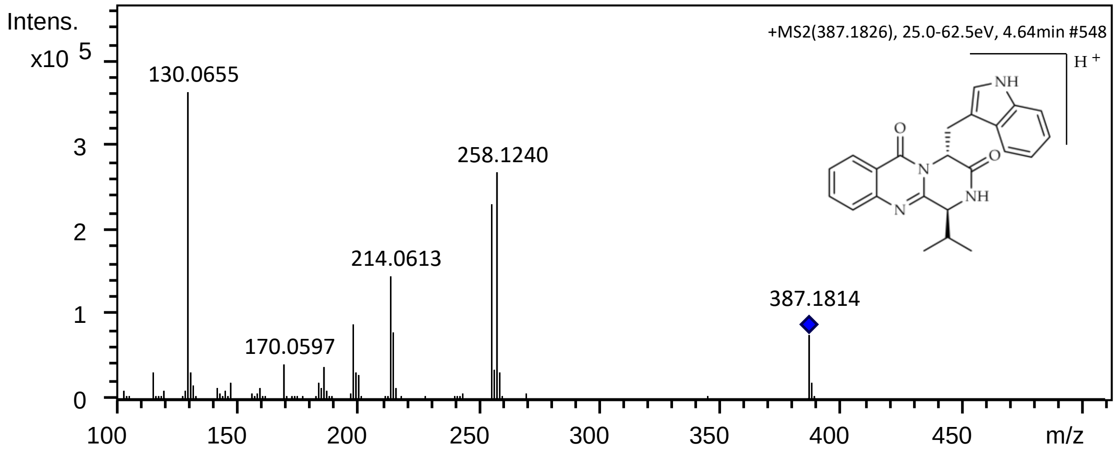

Figure S3. LC-HRMS/MS spectrum of fiscalin B (**1**), *m/z* 387.1826.

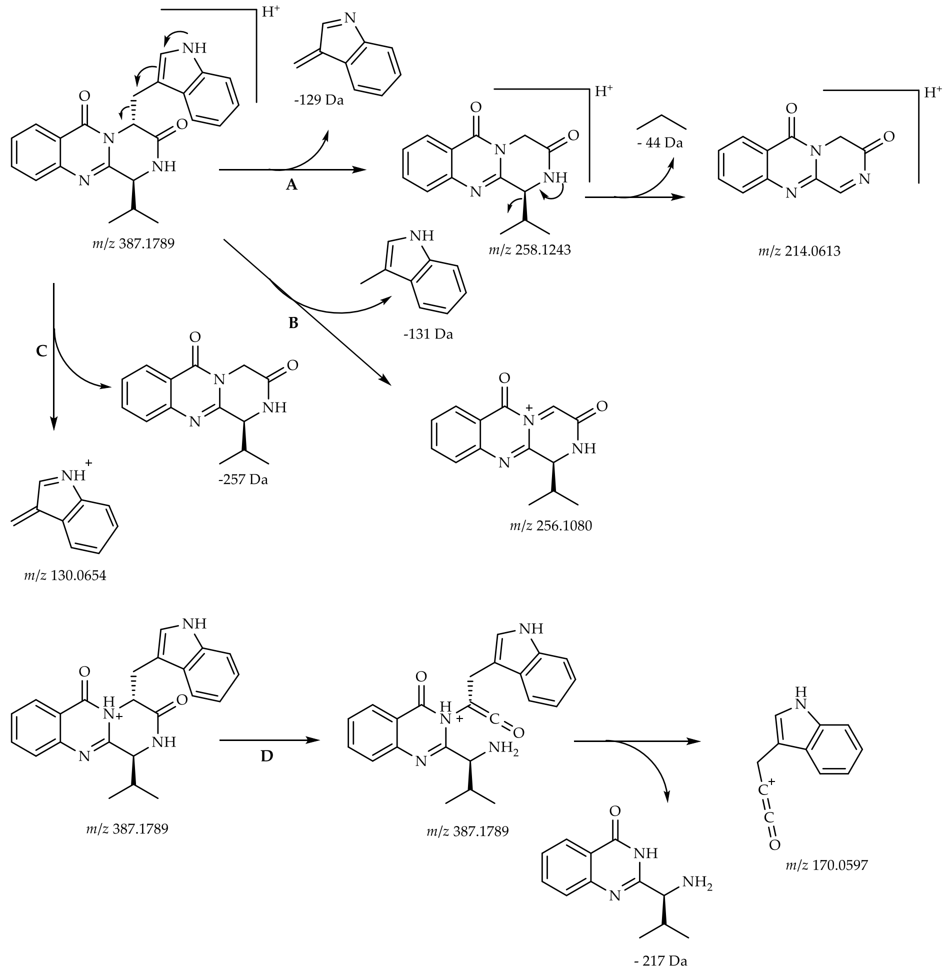

Figure S4. Proposed fragmentation patways of fiscalin B (**1**) in positive ion mode.

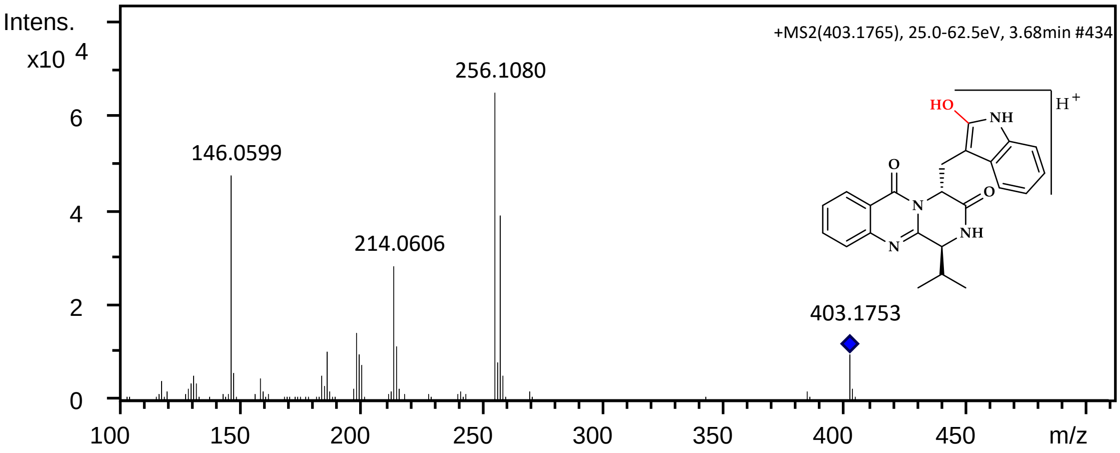

Figure S5. LC-HRMS/MS spectrum of M1, *m/z* 403.1765.

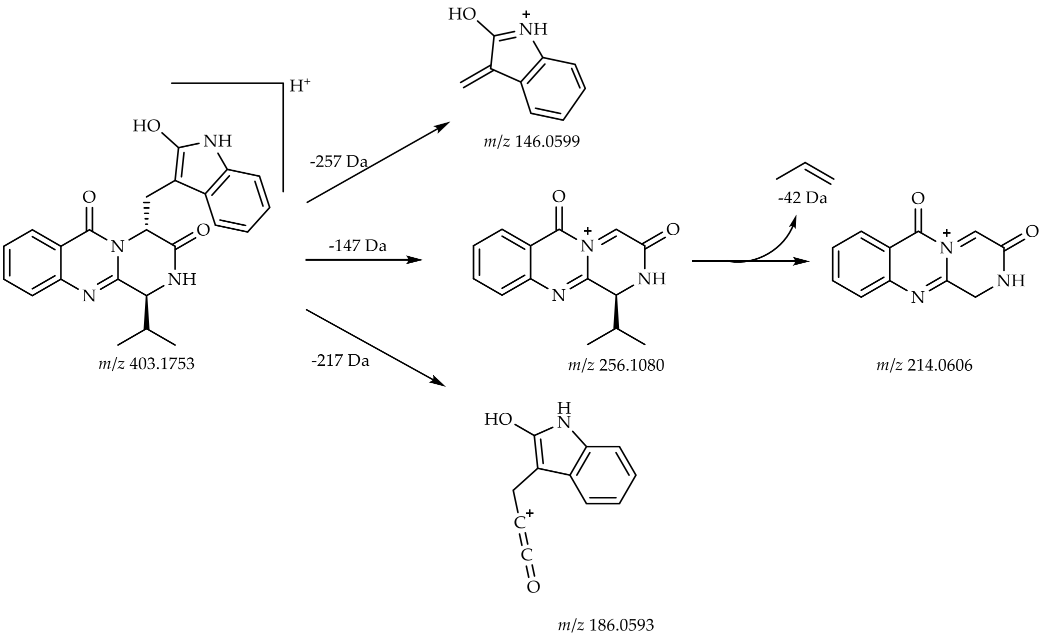

Figure S6. Proposed fragmentation pathways of metabolite M1.

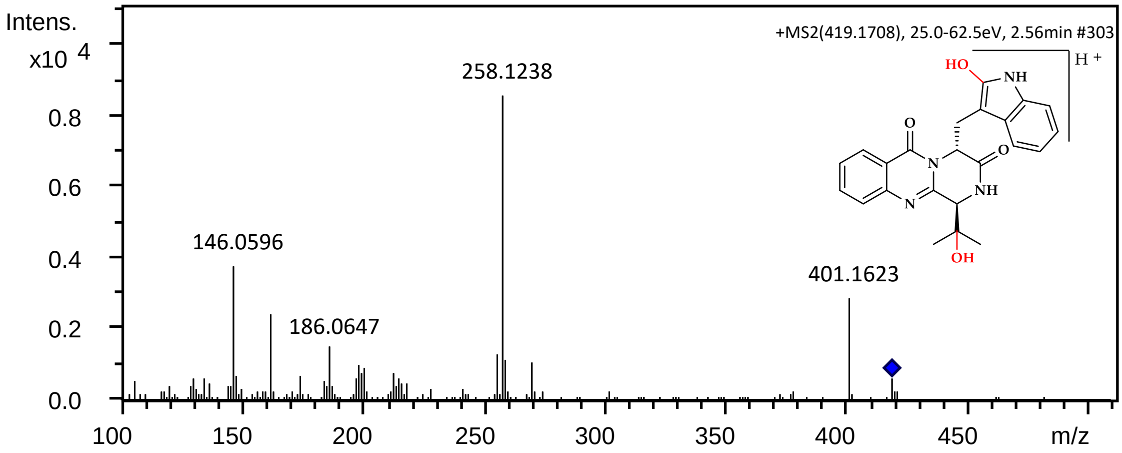

Figure S7. LC-HRMS/MS spectrum of M2, *m/z* 419.1708.

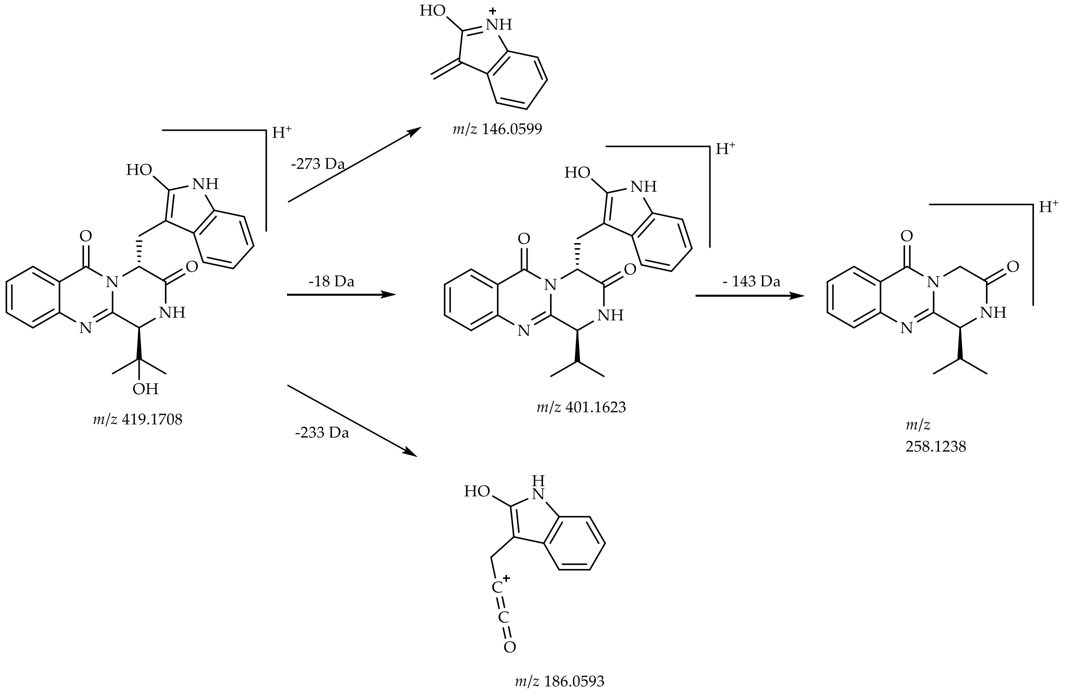

Figure S8. Proposed fragmentation pathways of metabolite M2.

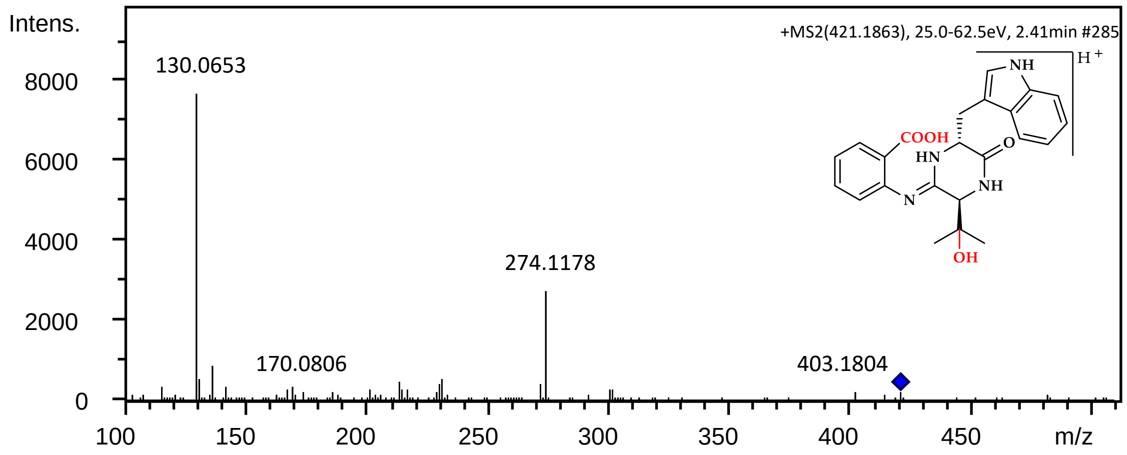

Figure S9. LC-HRMS/MS spectrum of M3, *m/z* 421.1863.

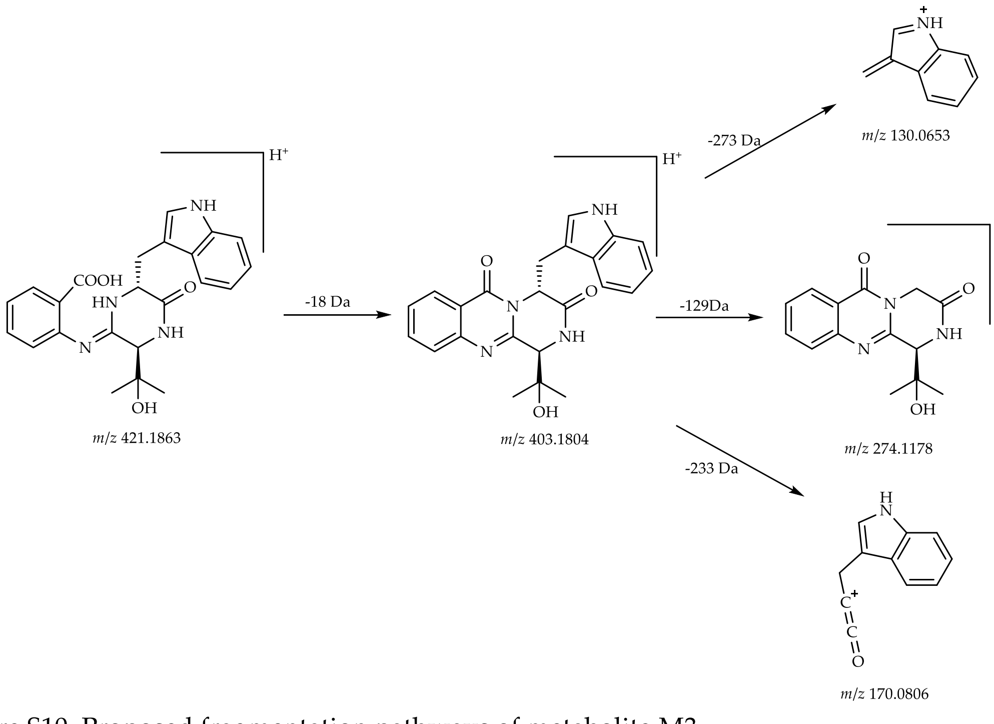

Figure S10. Proposed fragmentation pathways of metabolite M3.

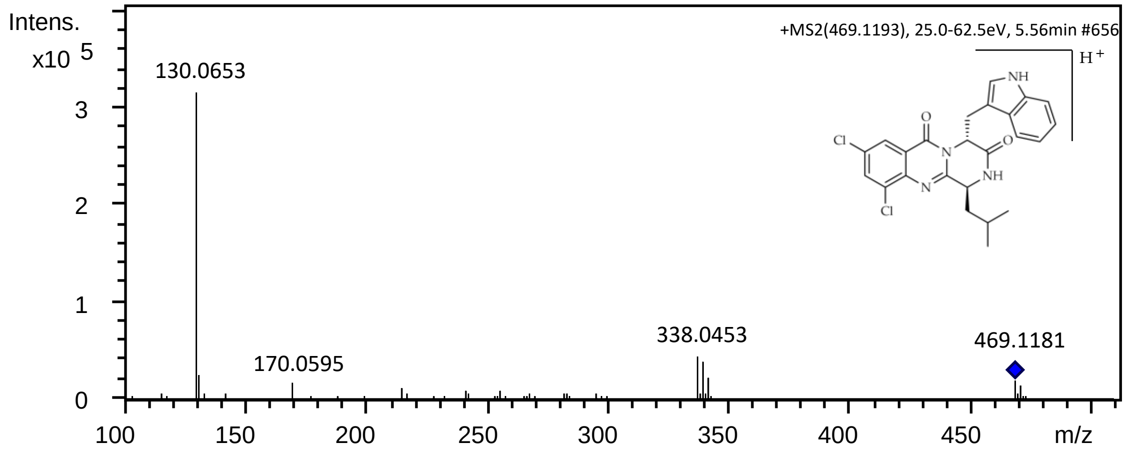

Figure S11. LC-HRMS/MS spectrum of fiscalin B chloro derivative (**2**), *m/z* 469.1193 in positive ion mode

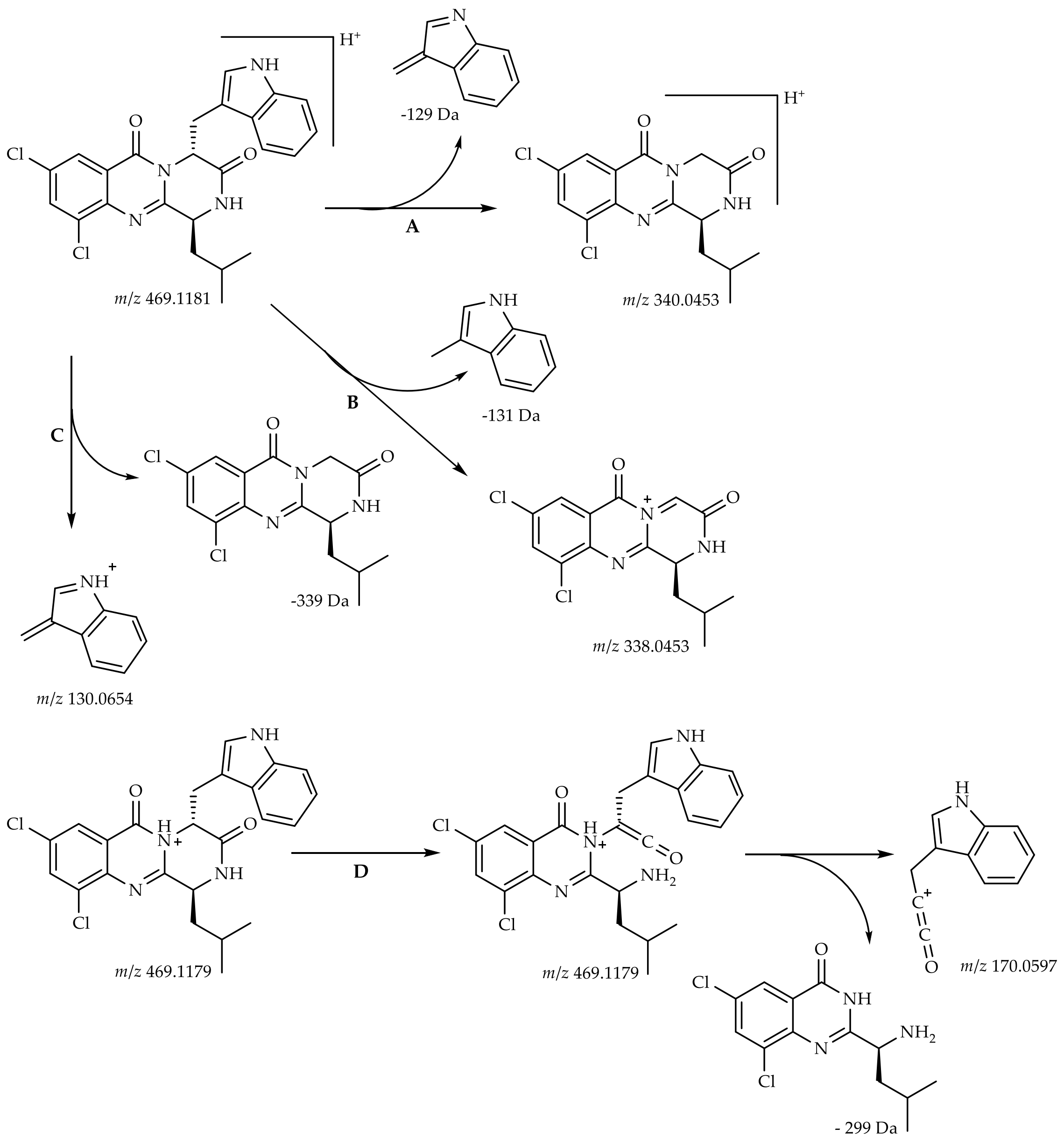

Figure S12. Proposed fragmentation patways of fiscalin B chloro derivative (**2**).

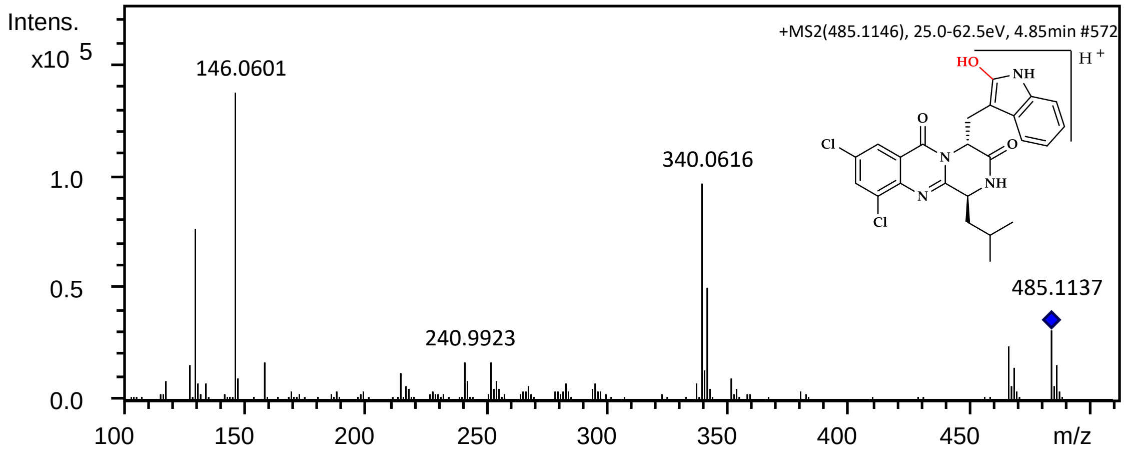

Figure S13. LC-HRMS/MS spectrum of M4, *m/z* 485.1146.

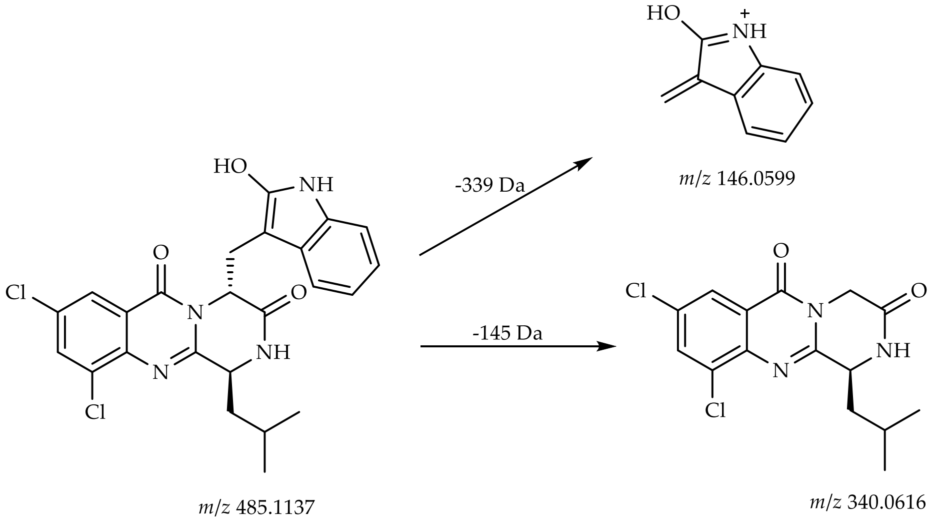

Figure S14. Proposed fragmentation pathways of metabolite M4.

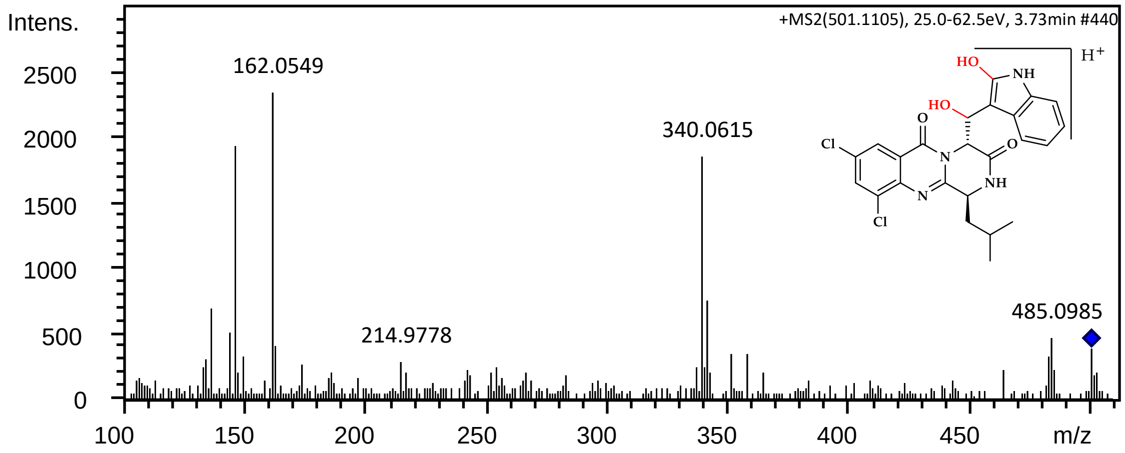

Figure S15. LC-HRMS/MS spectrum of M5, *m/z* 501.1105.

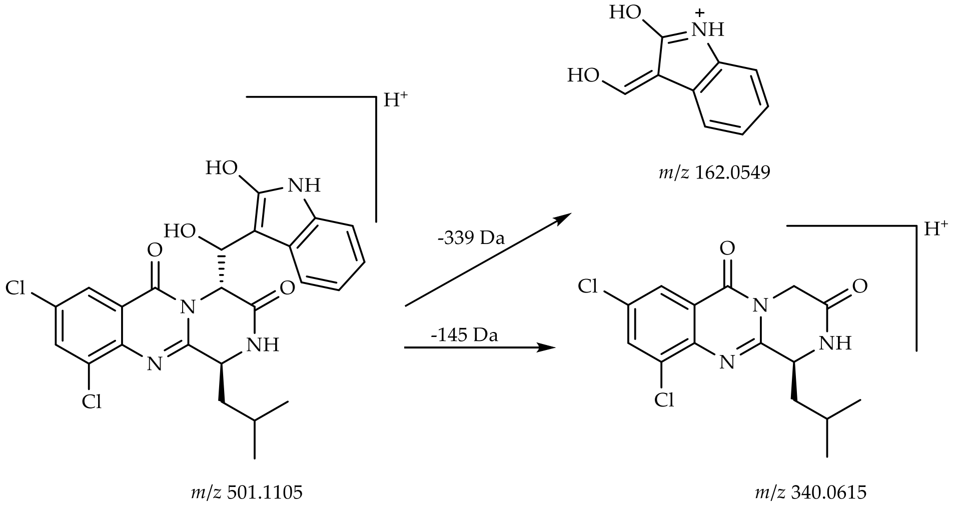

Figure S16. Proposed fragmentation pathways of metabolite M5.
